# Supplementary material for: Temperature dependence of Coulomb oscillations in a few-layer two-dimensional WS2 quantum dot
Source: Sci Rep. 2015 Nov 5;5:16113. doi: 10.1038/srep16113 (PMC4633606; doi:10.1038/srep16113)
Supplement: Supplementary Information [file srep16113-s1.pdf]

Supplemental Material for:

Temperature dependence of Coulomb oscillations in a few-layer two-dimensional WS_2 quantum dot

Xiang-Xiang Song,[1,2] Zhuo-Zhi Zhang,[1,2] Jie You,[1,2] Di Liu,[1,2]
Hai-Ou Li,[1,2] Gang Cao,[1,2] Ming Xiao,[1,2] and Guo-Ping Guo[1,2]

[1] Key Laboratory of Quantum Information, CAS, University of Science and Technology of China, Hefei, Anhui 230026, China

[2] Synergetic Innovation Center of Quantum Information & Quantum Physics, University of Science and Technology of China, Hefei, Anhui 230026, China

1. Estimation of E_C of the WS_2 quantum dot

In order to obtain the charging energy E_C precisely, we need to plot the Coulomb diamonds diagram in logarithmic scale. Fig. S1 shows the similar Coulomb diamonds diagram with Fig. 2(a), but in logarithmic scale.

The bias voltage V_{SD} tuned the Fermi level in the source (drain is kept ground), which opens a bias window for the electron to transport through the quantum dot (QD). We can obtain the charging energy E_C from the bias voltage we applied. As shown in Fig. S1, we can estimate that all of the Coulomb diamonds have the same charging energy E_C of approximately 0.75 meV.[S1]

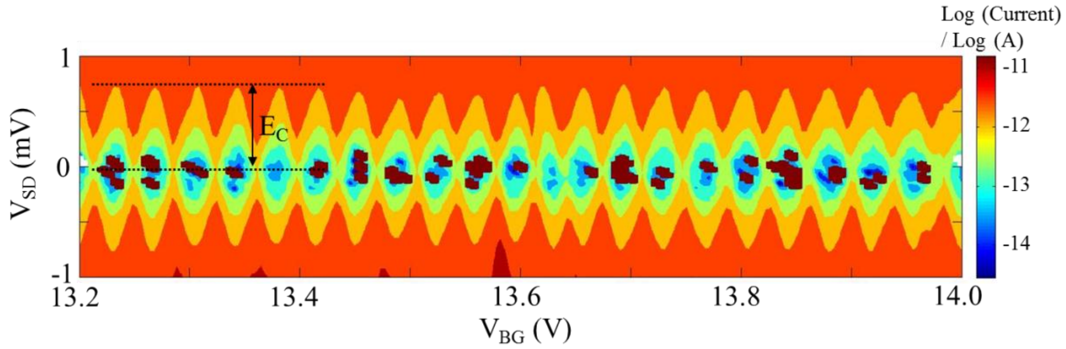

Figure S1. Logarithmic plotting of Coulomb diamonds diagram shown in Fig. 2(a).

2. Quantum-dot-like behavior of impurity trap in WS_2 device

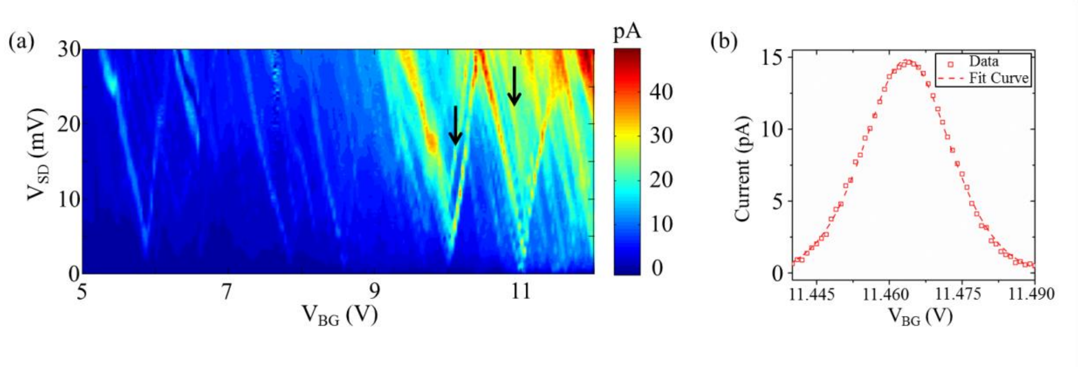

Figure S2. (a) A series of Coulomb diamonds found at larger V_{SD} considered to be the result of an impurity trap. (b) A typical Coulomb peak of the impurity trap fitted with cosh(x) (red dashed line).

Fig. S2(a) shows the Coulomb diamonds found at larger V_{SD} values. This series of Coulomb diamonds has a charging energy E_C larger than 30 meV and exhibits more than one period. The effective "dot" radius is estimated to be 10 nm. In addition, this series of Coulomb peaks is almost independent of the voltages applied to the top gates. All of this is evidence of an impurity trap that behaves like a quantum dot (QD).[S2] Fig. S2(b) shows a typical Coulomb peak of the impurity trap. Because the "QD" radius is 10 nm, a much larger single particle energy, ΔE=2.6 meV, is obtained. This value is

consistent with the experiment because evidence of the presence of an excited state is observed, indicated by the black arrows in the Coulomb diamond diagram (Fig. S2(a)). It is estimated that k_BT~50 μeV, suggesting that the Coulomb peaks of the impurity trap are in the quantum regime ($k_B T \ll \Delta E$). A $\cosh(x)$ fit is used to fit the Coulomb peak, given by the red dashed line in Fig. S2(b).

3. Temperature dependence of impurity trap Coulomb peaks

Similar to the WS_2 QD, we investigate the temperature dependence of two typical peaks of the impurity trap (labeled peaks 4 and 5). To exclude the influence of the QD, a bias voltage of V_{SD}~1 mV is applied.

Fig. S3(a) shows the peak height as a function of temperature. The height of the Coulomb peak decreases linearly with increasing temperature, which is the signature of a Coulomb peak in the quantum resonant tunneling regime.[S3] The temperature dependence of the full-width-at-half-maximum (FWHM) of the Coulomb peak is shown in Fig. S3(b). Different from the predicted linear dependence,[S3] the FWHM remains almost constant from 290 mK to 1 K. This also can be explained by the standard semiconductor QD theory.[S1] Because it is necessary to avoid the influence of the QD when detecting the current of the impurity trap, a typical dc bias of 1 mV is applied. This voltage V_{SD} gives an energy of approximately 1 meV to the "dot", which corresponds to an energy of 11.6 K. Under these circumstances, the temperature can no longer dominate the FWHM of the Coulomb peak. Such bias voltage also contributes to the different range of the Coulomb peaks obtained from the QD and the impurity trap.

Although the "QD" is formed owing to the impurity trap, the behavior of the Coulomb peaks is also in agreement with standard semiconductor QD theory.

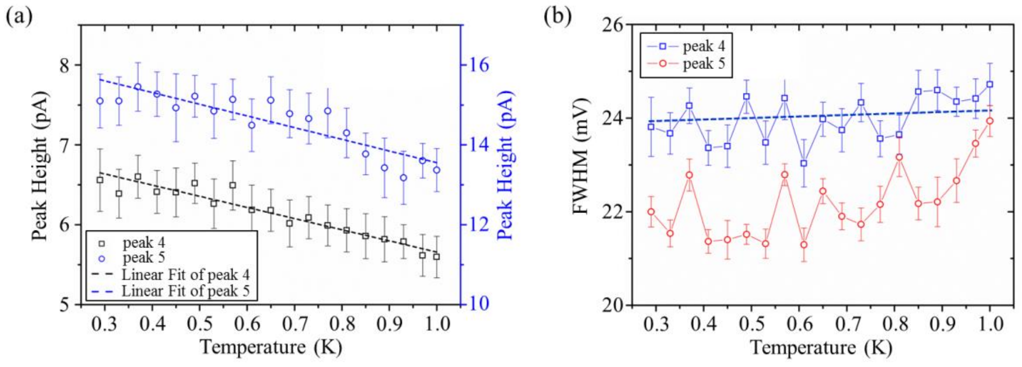

Figure S3. (a) The peak height of two typical Coulomb peaks (labeled peaks 4 and 5) of the impurity trap as a function of temperature, including the linear fits (dashed lines), exhibiting a linear decrease with temperature. (b) Temperature dependence of the FWHM of the two Coulomb peaks in (a) (labeled peaks 4 and 5).

REFERENCES

S1. L. P. Kouwenhoven, C. M. Marcus, P. L. Mceuen, S. Tarucha, R. M. Westervelt, and N. S. Wingreen, Electron transport in quantum dots. *Kluwer Series*, **E345,** Proceedings of the NATO Advanced Study Institute on Mesoscopic Electron Transport, 105-214 (1997).

S2. M. Sanquer, M. Specht, L. Ghenim, S. Deleonibus, and G. Guegan, Coulomb blockade in low-mobility nanometer size Si MOSFET's. *Phys. Rev. B* **61,** 7249 (2000).

S3. C. W. J. Beenakker. Theory of Coulomb-blockade oscillations in the conductance of a quantum dot. *Phys. Rev. B* **44,** 1646 (1991).
